# The truth is in the distribution

**DOI:** 10.7554/eLife.21723

**Published:** 2016-10-19

**Authors:** Indira M Raman

**Affiliations:** Department of Neurobiology, Northwestern University, Evanston, United States

**Keywords:** careers in science, women in science, living science

## Abstract

There may be as many ways to think about the experience of women in science as there are women in science. **Indira Raman** offers one perspective.

In my eighteen years as a professor of neurobiology I have often been asked to speak as a scientist because I am a woman, but this is the first time I have been asked to speak as a woman because I am a scientist*. The topic of women in science is complicated because the focus varies so widely for different people. For some, the primary issue is how to make a scientific career compatible with child-rearing; for others, it is how to get credit as an intellectual force, or how to deal with direct sexual harassment, or how to respond to the low ratio of female to male academic scientists, or how to react to pay inequities. Many ongoing efforts to address these issues are centered upon modifying the structures that form the institution of science and frame its practice, with the idea that these changes will confer power, in its most positive sense, upon women. Such work is indispensable. Yet maximizing the good effects of such endeavors depends on parallel efforts by those of us who inhabit these structures: we have to assume the power – and the associated responsibility – that already lies within our grasp. It is this complementary work that I wish to discuss.

With a nod to those colleagues who default to delivering their research seminar no matter what they are asked to talk about, I will adopt the same approach, with the hope that it will provide a scaffold for thinking about women as scientists. In several branches of biology, and certainly in my own field of cellular neurophysiology, many experimentalists are reaching the conclusion that the key to biological principles lies not simply in the mean value of any parameter, but in the variance. The variance turns out to be more than just noise; often, it is the essence of the code. For example, groups of neurons that were long seen as homogeneous emerge as heterogeneous: although they share common properties, each has its own character. From interactions among these related yet distinguishable entities, the elegant and intricate abilities of the system emerge. Likewise, with people – men, women, brown, light, native, and immigrant – a stereotype may offer a description of a mean or even a mode, but no generalization captures the core features, or predicts the path, of any individual. Instead, the truth is dispersed throughout the distribution.

My highly personal perspective on this point emerged in the early phases of my career, when I could go through whole swaths of the working day without actively thinking about being female. In fact, what I still enjoy most about doing science is that it can lift me, temporarily, out of the strictures of social norms into the beautiful world of physical reality that *isn’t* about the self. Nature’s laws – her symmetry and her chaos, her principles and her evolution – belong to everyone. They reveal themselves to anyone who searches honestly. At social gatherings with non-scientists, I invariably realize anew how much freedom science affords us women to become the best of ourselves. And that, I think, is largely what each of us is striving for – the excitement of being permitted to venture unhindered into unexplored intellectual territory, which necessarily confers a uniqueness of endeavor onto each of us, and the pleasure of being recognized for our discoveries. Doing science allows us the luxury of finding our own private places in the distribution.

To me, the issues of women and science break down into three broad categories: how others see us; how we see others, and how we see ourselves. In each case, a tension lies between perceiving people as stereotypes and viewing them as individuals with a marvelous palette of idiosyncrasies.

The impartial house of scientific truth is a delightful haven for those of us who have never fit well into the group.

The first category, of how others see us and whether stereotypes dictate how we are treated, receives a lot of attention. Here, I will revert to the personal and assert that some bad things have happened to me in this field because I am female – or to be precise, those bad things correlate strongly with being female. I need not list them; most people know what they are. While acknowledging them, however, I must also acknowledge that many good things have happened to me likely because I am female. Still — and I think it is important to emphasize this — only a small fraction of my total scientific experience seems tightly linked to being female. The evidence is that I have met many men who have overcome their own versions of the hurdles that I think of as having shaped my scientific experience. More exactly, I find that, although the details of each person’s trajectory are unique, *everyone* is navigating the constraints placed upon them by their physical selves – for example, their height, weight, hue, looks, accent, or vocal timbre – while contending with the fundamental challenges of doing science.

**Figure 1:**
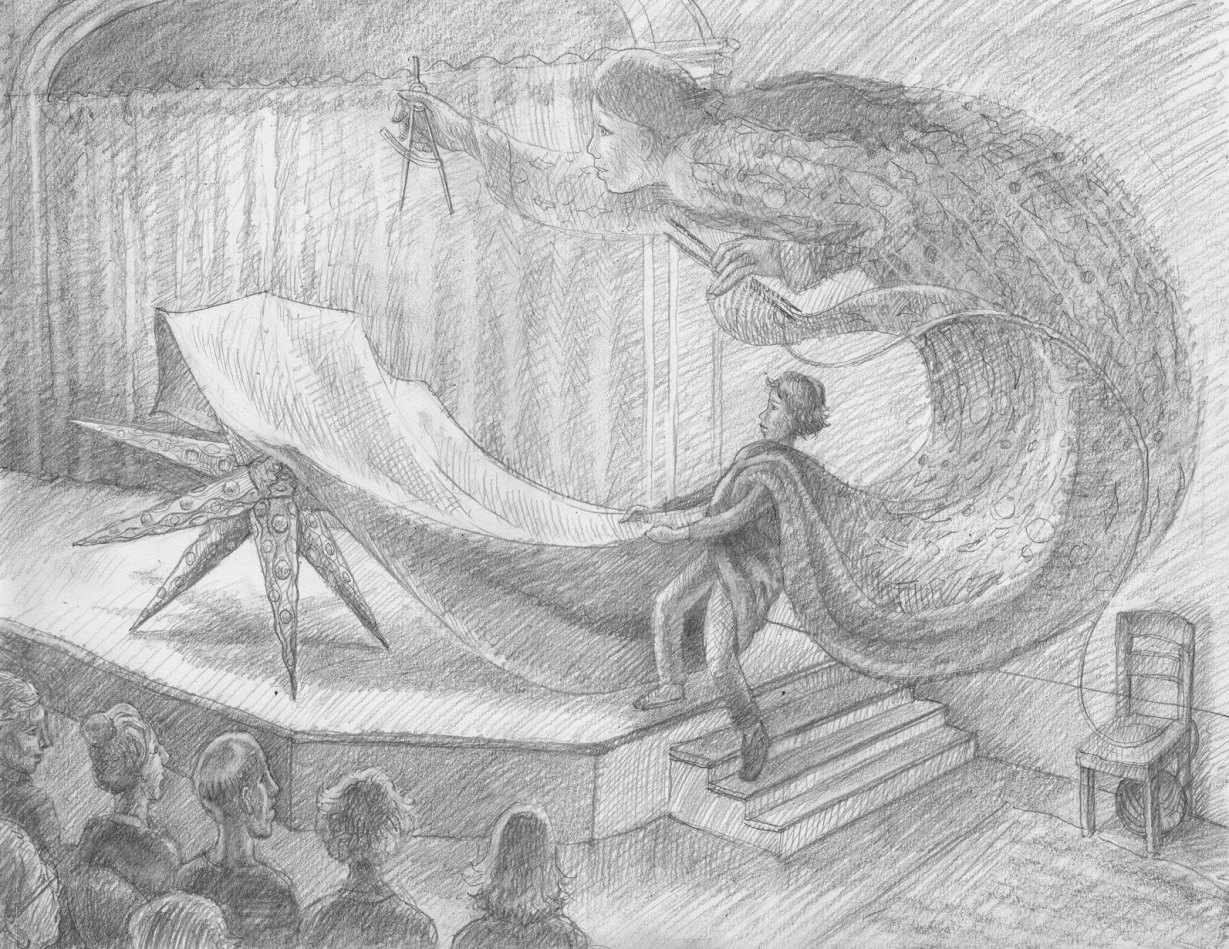
You walk on stage as a woman, and you walk off stage as a scientist.

Illustration: Ben Marder

Focusing on the female-linked events, however, we can start with the good parts. We are lucky enough to live in an age, unlike our female predecessors, in which many institutions not only are willing to accommodate women but also will go out of their ways to do so. Oddly, this often generous inclusiveness can re-formulate the old question of whether female scientists are taken seriously – as a woman, sometimes you can’t help wondering whether you were invited to give a keynote talk (or awarded an honor, or appointed to a committee), just so that someone can confirm an acceptable gender balance. The way I have found to deal with this uncertainty is internal: you walk on stage as a woman, and you walk off stage as a scientist. When you give intelligently and sincerely of yourself to the individuals that compose the judging crowd, you stop being the stereotype and become the person you are. It’s liberating.

Doing science allows us the luxury of finding our own private places in the distribution.

Admittedly, however, it is not always easy, which brings us to the female-linked bad experiences. Some hostile people lurk out there, and that fact is inescapable. There is an ecological niche for them, and they are not going away. But even if someone is best described by a harsh negative word, it is actually bad science to conclude that his actions are an inevitable result of his masculinity. Some men do impede women’s progress in science, but many, perhaps most, women in science can think of at least one male scientist who helped them substantially along their way. Diversity among men is as real as diversity among women, and many men are allies in the efforts to advance the causes of all people in science. And that, too, is liberating: a man engaging in bad behavior becomes a specific case rather than a representative of a conspiracy.

Some men do impede women’s progress in science, but many, perhaps most, women in science can think of at least one male scientist who helped them substantially along their way.

And that brings us to second category, of how we see others. I think one thing that causes pain to many women (and men) in science comes from the mental image that successful scientists conform to a Platonic ideal, which is often a blend of Leonardo da Vinci, Isaac Newton, and Albert Einstein, transformed into a modern, English-fluent man. He was born to privilege, pored over microscopes and matrix algebra as a youth, and dominated all his peers before he stormed noisily or stalked grimly or sailed confidently into science. I don’t know exactly who this person is, but I do know that a lot of aspiring scientists assess themselves by the differential between him and themselves.

In this context, it is worth noting that many men, too, struggle with the sense that they cannot bring themselves to be as aggressive as they think being a scientist requires; I have listened to several such accomplished trainees and colleagues speaking emotionally in my office. That is one privilege of being the female that I am: the peculiar spot that I occupy within the distribution makes me less of a competitor, and consequently, a remarkable range of men and women have dared reveal to me something of who they really are. By coming to know the richness of their specific personalities, my own assumptions disintegrate. Their variance renders me more humane.

And that variance, along with the ability coupled to it, demonstrates that *there are* many ways to succeed in science. Aggression is indeed one of them, as is opportunism, or dogged single-mindedness. But so is merit. None of these categories are mutually exclusive, and, importantly, all are independent. Merit alone can often be enough to drive scientific success, but there is no prefabricated formula for becoming meritorious. You have to think and do and train yourself and make use of your resources and derive inspiration from all kinds of people: male, female, self-like, other-like. Anyone who shows you a wise way of doing or being can be a role model. You do not have to be exactly like them; you don’t even have to want to be exactly like them. Almost no one is likely to attain complete coherence as a scientist or as a person. We may be outstanding in some areas and still have to compensate for weakness in others. Science is not about conforming to an ideal, masculine or feminine, but instead relies on the diversity of perspective that gives rise to insight. The individuals do not derive identity from the group; the group is defined by the identity of its component individuals. Or, as I phrase it to myself, the point is not whether I think like other scientists do; it’s that one scientist – me – thinks like I do. Being scientists means self-assessing honestly, by giving ourselves rational credit for our achievements as well as reasonable correctives for our errors. Doing so is a power and a responsibility that we take upon ourselves.

Science is not about conforming to an ideal, masculine or feminine, but instead relies on the diversity of perspective that gives rise to insight.

And that has led us to the third category, of how one sees oneself. My conversations give me the impression that many women see themselves as limited by a stereotype defined by traits that hinder success, such as nervousness and lack of confidence. Many women (and men) find it distressing that they get nervous before any public performance – from submitting a manuscript to delivering a seminar – and they read within their own reactions a critique of their scientific worth. I maintain, however, that nervousness is underrated. When used properly, it can spur you to prepare, think carefully, reflect, practice, and ultimately do a fine job. Only when anxiety becomes paralytic does it become problematic. Otherwise, self-doubt need not be perceived as a handicap. In fact, it is central to the progress of science. The best work is not achieved through complacency.

One might argue, however, that the best work *does* depend on confidence – specifically, on people who have confidence in the work they do. But confidence (when it isn’t foolhardiness) is not a mystical quality, either innate or granted by a mentor. Confidence is just memory. It is the memory of having faced challenges and overcome them through your own efforts, frequently enough so that the awareness of having surmounted difficulties before makes your brain quite sensibly anticipate that you will do so again. The only way to gain confidence is to *do*, and then to observe your triumphs and failures, and to edit your behavior until the triumphs exceed the failures sufficiently often for the most logical prediction to be that of success. And your memory is, arguably, what confers individuality. That is the essence of what I have learned: by each developing a memory of doing science well, whatever the irregular trajectory to the outcome, we can each find our own place in the distribution. Only then can those of us who are female be not just women in science, but scientists.

## Footnote

*A version of this essay was delivered at the 2016 Gordon Research Conference on Synaptic Transmission as a talk for a “Power Hour” on empowering women in science. Some ideas overlap with those in Raman IM. 2014. How to be a graduate advisee. *Neuron* **81**:9–11.

